# Perinatal Women’s Views of Pharmacist-Delivered Perinatal Depression Screening: A Qualitative Study

**DOI:** 10.3390/ijerph192316295

**Published:** 2022-12-05

**Authors:** Lily Pham, Rebekah J. Moles, Claire L. O’Reilly, Stephen Carter, Camille Raynes-Greenow, Timothy F. Chen, Corina Raduescu, Sue Randall, Jacqueline Bloomfield, Clara Strowel, Andrea Murphy, David Gardner, Sarira El-Den

**Affiliations:** 1 The University of Sydney School of Pharmacy, Faculty of Medicine and Health, The University of Sydney, Sydney, NSW 2050, Australia; 2 Sydney School of Public Health, Faculty of Medicine and Health, University of Sydney, Sydney, NSW 2050, Australia; 3 The University of Sydney Business School, University of Sydney, Sydney, NSW 2050, Australia; 4 Susan Wakil School of Nursing and Midwifery, Faculty of Medicine and Health, University of Sydney, Sydney, NSW 2050, Australia; 5 College of Pharmacy, Faculty of Health, Dalhousie University, Halifax, NS B3H 4R2, Canada

**Keywords:** perinatal depression, women, pharmacy, pharmacist-delivered, screening

## Abstract

Internationally, 20% of women experience perinatal depression (PND). Healthcare providers including general practitioners and midwives are critical in providing PND screening and support; however, the current workforce is unable to meet growing demands for PND care. As accessible and trusted primary healthcare professionals, pharmacists could provide PND care to complement existing services, thereby contributing to early detection and intervention. This study aimed to explore perinatal women’s views of community pharmacist-delivered PND screening and care, with a focus on their attitudes towards and acceptability of PND screening implementation in community pharmacy. Semi-structured interviews with women (*n* = 41) were undertaken, whereby interview data were transcribed verbatim and then inductively and thematically analysed. Five overarching themes emerged; “patient experience with existing PND support and screening services”; “familiarity with pharmacists’ roles”; “pharmacist visibility in PND screening care”; “patient—pharmacist relationships” and “factors influencing service accessibility”. Themes and subthemes were mapped to the Consolidated Framework for Implementation Research. Findings highlight participants’ generally positive attitudes towards community pharmacist-delivered PND screening and care, and the potential acceptability of such services provided pharmacists are trained and referral pathways are established. Addressing perceived barriers and facilitators would allow community pharmacist-delivered PND screening and care to support existing PND care models.

## 1. Introduction

The perinatal period, the time during pregnancy and up to 12 months after delivery, can be a particularly vulnerable time for women [1]. Globally, perinatal depression (PND) affects 15–65% of women antenatally [2] and 16–19% of women postnatally [3]. Furthermore, poor maternal mental health is associated with negative outcomes including pre-term delivery; low birth weight and poor parent-child bonding [2,4,5].

In Australia, PND affects approximately one in five women, with 80% receiving some support or treatment [6]. Despite increases in Australian PND screening rates, one in five women do not receive mental health screening both antenatally and postnatally [7]. Similarly, data from one of the largest obstetric services providers in Minnesota (USA) indicate that 34.9% of eligible women are not screened for PND [8]. Populations at higher risk of PND, including older women and those reporting high emotional distress, are also less likely to be screened [7]. Perinatal mental health is a growing area of health concern with a two-fold increase in new callers to the Perinatal Anxiety and Depression Australia (PANDA) helpline since March 2020 [9].

PND screening increases mental health service access, referral rates and may improve emotional outcomes [10]. However, Australian PND screening rates do not meet national clinical practice recommendations endorsing universal screening [7]. Hence, more resources are required to complement existing infrastructure. The lack of adequate pathways to mental healthcare for perinatal women also needs to be addressed to reduce national disease burden [11]. A systematic review exploring PND screening recommendations from member countries of the Organisation for Economic Co-operation and Development highlighted that most endorsed PND screening [12] and that there is a need for a broad range of healthcare professionals to contribute to early detection and intervention [12].

Pharmacists are amongst the most accessible and trusted healthcare professionals [13] who interact regularly with consumers, including perinatal women. While PND screening recommendations rarely specify pharmacists’ roles [12], some Australian publications have included pharmacists explicitly [14] and potentially more broadly when encouraging “all health professionals providing care to women in the perinatal period” to be involved in PND screening [15]. The emerging literature demonstrates that pharmacists self-identify as being ideally placed to identify and refer women presenting with PND symptoms; however, they recognise their lack of familiarity with PND referral pathways and the need to be further trained in this area [16]. Psychometrically validated questionnaires intended to measure pharmacists’ PND knowledge [17], attitudes and screening acceptability [18] have been developed. Furthermore, the need to explore the acceptability of pharmacist-delivered PND screening by stakeholders has been acknowledged [19].

Studies have investigated women’s views towards nurse-led postpartum depression screening and counselling [20], the public’s acceptability of perinatal mental health screening [21], as well as physicians’ and paediatricians’ attitudes towards providing postpartum depression screening [22]. However, there is a lack of research exploring women’s attitudes towards pharmacist-delivered PND screening and care. Implementation science bridges the gap between research and practice by exploring how evidence-based findings can be incorporated into routine healthcare practices [23]. The application of a framework such as the Consolidated Framework for Implementation Research (CFIR) can be used to explore perspectives of key stakeholders, including perinatal women, relating to the potential implementation of pharmacist-delivered PND screening and care [24]. Although the CFIR has frequently been applied when investigating clinical and operational stakeholder perspectives [25,26,27,28], the framework has been increasingly used in studies exploring patient perspectives [29,30,31,32,33,34]. Recently, the CFIR has been applied to explore patient perspectives on health service implementation [35,36] and pharmacy-delivered services [37,38]. While consideration of consumer perspectives when implementing health services was previously uncommon [39], this has become increasingly recognised as crucial to healthcare service planning and delivery [40,41]. This study therefore aims to explore perinatal women’s views of community pharmacist-delivered PND screening and care, with a focus on attitudes towards and acceptability of PND screening implementation in community pharmacy.

## 2. Methods

An interdisciplinary research team consisting of a perinatal epidemiologist, business academic, pharmacists, Mental Health First Aid (MHFA) instructors and mental health researchers with expertise in mental health, community pharmacy-based services, perinatal health and business operations conceived the study, developed the interview guide and contributed to the interpretation of the findings. The Standards for Reporting Qualitative Research (SRQR) where used as a guideline for this study (Appendix A).

### 2.1. Recruitment

A recruitment advertisement was disseminated through websites, social media and emails of state and territory mothers’ and parents’ groups, from September to November 2020. Local community pharmacies known to the research team were contacted using publicly available information twice to display advertisements. The research team also disseminated the advertisement through social media platforms (i.e., Facebook, LinkedIn, Twitter). Participants who expressed interest in participating were contacted for an interview; non-response after three attempts was not followed-up further.

### 2.2. Participant Eligibility

Participants were eligible for inclusion if they were pregnant or up to twelve months postpartum at the time of the interview, spoke English and were Australian residents.

### 2.3. Interview Guide

A semi- structured interview guide developed by the research team was informed by study aims, a systematic review exploring PND screening acceptability [19] and a questionnaire measuring attitudes towards and acceptability of PND screening among pharmacists [18] (Appendix B). The interview guide included closed and open-ended questions to collect demographic data including participant age, multiparity, state or territory of residence, as well as women’s personal experiences with PND screening, relationship with pharmacists and awareness of PND. The interview guide also explored constructs such as usefulness, openness, comfortability, and acceptability regarding pharmacist-delivered PND screening and care. The proposed PND service outlined in the interview guide included screening with the Edinburgh Postnatal Depression Scale (EDPS), evaluation of EPDS scores and referral to the general practitioner (GP), if necessary.

### 2.4. Data Collection

Semi-structured interviews were conducted via telephone or video conferencing platforms. Interviews were audio-recorded and transcribed verbatim for analysis. The interviewer (LP) had previous experience in conducting, interpreting and analysing qualitative interviews for publication [42]. LP was also MHFA-trained and could provide support if participant distress was recognised. Interviews were conducted between October 2020 and February 2021 until data saturation was reached. The University of Sydney Human Research Ethics Committee (Project number: 2020/584) approved the study.

### 2.5. Data Analysis

Data analysis was conducted between September 2021 and January 2022. One researcher (LP), a community, hospital and research pharmacist whose research focuses on perinatal depression screening in community pharmacies led the qualitative analysis in NVivo [43]. LP immersed herself in the data by conducting all interviews, listening to audio-recordings and reading transcripts multiple times. Data were initially inductively coded to determine overarching themes and subthemes related to barriers and facilitators towards pharmacist-delivered PND screening, using a combination of semantic and latent approaches. Participant quotes categorised under each theme and corresponding subtheme/s were then deductively coded to the CFIR which presents five domains associated with effective innovative implementation [44]. Domain definitions were adapted from Damschroder and Lowery, as well as Safaeinili, Brown-Johnson, Shaw, Mahoney and Winget [25,44,45], then applied to women’s perceptions of pharmacist-delivered PND screening and care (Table 1). Adapted domain definitions were used to guide the mapping of theme and subtheme/s to the CFIR domain and associated construct. A random 20% sample (*n* = 8) of transcripts were independently coded by CS who considered themes and subthemes, mapping them to CFIR to ensure coding reliability and consistency [46]. CS is not a pharmacist but has experience in perinatal mental health education research. Both coders discussed emerging themes, subthemes, domains and constructs, with consensus reached for discrepancies.

Qualitative analysis was further consolidated by regular discussion between LP and three research team members (SED, RM, COR). Barriers and facilitators to community pharmacist-delivered PND screening were considered when synthesising the results, where relevant.

## 3. Results

### 3.1. Participant Recruitment and Characteristics

Of the 16 community pharmacies contacted, three pharmacies promoted the study. Six mothers’ groups and one parents’ group across six Australian states also promoted the study. A total of 58 participants expressed interest, of which 41 were recruited and consented to participate (Table 2).

### 3.2. Qualitative Analysis

Women interviewed were generally familiar with the term “*perinatal depression*” and those who were not were familiar with the term “*postnatal depression*”. Five overarching themes describing participants’ acceptability and views of, as well as barriers and facilitators to, pharmacist-delivered PND screening and care emerged: “patient experience with existing PND support and screening services”; “familiarity with pharmacists’ roles”; “pharmacist visibility in PND screening care”; “patient–pharmacist relationships” and “factors influencing service accessibility” (Appendix C).

### 3.3. Theme 1 Patient Experience with Existing PND Support and Screening Services

Two subthemes “recognised sources of perinatal mental health support” and “previous experience with PND screening” were derived from the theme “patient experience with existing PND support and screening services.” Subtheme “recognised sources of perinatal mental health support” was mapped to the CFIR domain “inner setting” and associated constructs, “readiness for implementation’, ‘culture’, as well as “networks and communications”. Subtheme “previous experience with PND screening” was mapped to CFIR domain, “outer setting” and its construct, “patient needs and resources”, as well as domain, “inner setting” and its construct “implementation climate”.

### 3.4. Subtheme 1.1: Recognised Sources of Perinatal Mental Health Support

Women were generally comfortable in seeking support when experiencing depressive symptoms, citing family, friends, healthcare professionals, online resources and telephone support services as support sources. Participants felt that the healthcare supports accessed often depended on the woman’s stage, in that “*if it was during the pregnancy or in that first six weeks postpartum, I would have spoken to my obstetrician because that’s who I had the most connection with during my pregnancy… after that, it would have been my GP*” [ID36].

Obstetricians were sometimes preferred because they “*understand, they’ve seen a lot of women going through the same thing…they’ll be giving you more reassurance, could probably prescribe you medication if required…guide you to the other channels that you can explore and maybe activities… that could improve your mood*” [ID9]. Generally, women reported having a “*good relationship with the GP… they’ve probably seen a lot of cases, are able to help you identify what’s going on, and have the right tools to assess that*” [ID27]. Women also felt that “*it was easiest to get [an appointment] in with her [the GP] first*” [ID30] as opposed to specialists including obstetricians. Child health nurses were also an access point for perinatal mental health support for “*advice about anything to do with my baby and are also aware of postnatal depression… they keep an eye out on those kinds of things as well*” [ID31]. On the other hand, PND screening by a child health nurse was found to be “*not super useful*” in some instances, as post-screening advice to simply “*go talk to someone*” made it hard for women to “*find the time to get the referral*”, emphasizing the importance of ensuring women had a post-screening “*plan… that’s actually realistic*” [ID33].

Online resources including parent’s groups, PANDA and Beyond Blue Australia were also cited as sources of support and “*reassurance… like what my experience is… is it normal?*” [ID3]. Advantages of telephone services included that they were “*anonymous and… if I needed help, I can just call one and ask for advice*” [ID10]. Parent’s groups were also recognised as support sources because other parents would be “*going through something either similar or something that they might have gone through in the past and they can share their experience or their advice*” [ID18]. However, online resources and hotlines were recognised as potentially unreliable, in that advice found online or from mothers’ groups “*isn’t always correct*” and that seeking help from professionals was preferable “*because they specialise in depression*” [ID32]. Nonetheless, some women explained that they would not proactively seek support “*I don’t think I would actively go and say I’m feeling pretty low. It’s more when people ask me, I would then sort of start talking a bit about it*” [ID24], highlighting the need for healthcare professionals to take on proactive roles.

Some women demonstrated an awareness of referral pathways, noting that they would first see the “*GP and then, get referred to counselling, a psychologist…because that’s just a standard process that I’m aware of*” [ID38]. For women with lived experience of depression who reported that they were already in contact with psychologists or counsellors, they would “*either contact them [psychologists] directly or speak to my GP… because it’s [a] pre-existing [condition]… this particular GP has been quite good with referring me on and doing my mental healthcare plan… there’s that trust that they’ll take it seriously*” [ID34]. Nonetheless, some women were not aware of professional support options and lacked social support. This included immigrants who explained that they “*don’t have my family close by and I just haven’t really talked [about] personal feelings… I would love to reach out with anybody professionally to talk about things like this… I’m not sure who I could reach*” [ID16].

### 3.5. Subtheme 1.2: Previous Experience with PND Screening

Most women reported being screened for PND during a perinatal healthcare appointment. Screening was completed individually by the woman or with a midwife, GP, nurse or obstetricians. Screening was self-reported by women to take less than one minute to 30 min. Women felt that PND screening during healthcare appointments was “*a normal part of the process*” [ID37] and were “*grateful that it felt like people were looking out for me*” [ID21]. Although several women expressed uncertainty by being “*slightly nervous about what the end result is going to be*” [ID23], many saw “*the real importance in it*” [ID14]. However, some reported only being screened once, either during pregnancy or in the postpartum period whilst others reported not being screened at all.

Women highlighted that time pressure can impact patient comfort, explaining that “*screening is a good tool, but it depends on how you use it. For example, I think in that booking visit, the midwife rushed things a bit. So… I don’t feel comfortable being 100% honest with how I feel*” [ID35]. Participants’ previous experiences with hotlines made some feel “*policed*” and “*very trapped by their safety action plan*” [ID15]. As a result of these experiences, some women reported that pharmacists “*will be a great resource because right now… you would have to jump through hoops to voice that you have an issue.*” [ID15]. The benefit of pharmacist-delivered PND screening was recognised by women who noted that “*a lot of women don’t want to start that [conversation] themselves. There’s often that fear of, ‘I’m going to be judged for not coping,’… so it’d take a lot of the pressure off of women to get that help because it’s already been offered, rather than them having to go to somebody, which might not happen for them*” [ID32]. Women’s preference to be approached by accessible healthcare professionals such as pharmacists for PND screening rather than proactively seeking help may facilitate the implementation of pharmacist-delivered PND screening.

### 3.6. Theme 2: Familiarity with Pharmacists’ Roles

Two subthemes “awareness of pharmacist-delivered health services” and “pharmacists’ roles in mental health” were derived from the theme of “familiarity with pharmacists’ roles.” Both subthemes were mapped to CFIR domain “characteristics of individuals”. However, subtheme, “awareness of pharmacist-delivered health services” was mapped to the construct “knowledge and beliefs about the intervention”, whilst the subtheme “pharmacists’ roles in mental health” was mapped to constructs, “knowledge and beliefs about the intervention” and “individual identification with the organisation”.

### 3.7. Subtheme 2.1: Awareness of Pharmacist-Delivered Health Services

While women recognised pharmacists’ roles ranging from blood pressure monitoring, vaccinations, baby-weighing, lactation consultations, to midwife and community nurse check-ups, most felt that a pharmacist was “*not traditionally someone that you would go for mental health support*” [ID5]. Nonetheless, women considered pharmacist-delivered screening to be a good idea and were open to pharmacists’ advice, provided that pharmacists were part of the healthcare team, trained in perinatal mental health, had trusting relationships with women and could offer private consultations: “*I think [it’s a good idea] as long as they’re [pharmacists] trained to do it… mental health isn’t spoken about enough… I think any conversation, any support that can be given… is a good idea because it shouldn’t be a taboo subject*” [ID5].

### 3.8. Subtheme 2.2: Pharmacists’ Roles in Mental Health

When pharmacist-delivered PND screening was suggested, some participants noted that they “*would never think to go to the pharmacist, to seek advice or be screened*” [ID26] for PND. There was a perception that pharmacists’ roles were confined to dispensing medication, medication safety and related services. Women questioned whether pharmacists’ education enabled them to provide mental healthcare “*my understanding of pharmacists is that they are more close to medications… I just don’t know… if their training is adequate for them to give you [mental health] advice*” [ID1]. The lack of consideration of pharmacists’ roles in mental health is a barrier to pharmacist-delivered PND screening implementation.

Women recognised that pharmacists should have a role in mental healthcare as “*anybody in the health profession has a certain duty of care when it comes to a person’s health, whether that’s mental or otherwise… the more widely available that we can make support for… mental health, the better*” [ID5]. Women suggested that pharmacists’ roles in this area could include facilitating access through “*linkage to other services*” [ID5]. However, women reflected that they were “*not sure what services they [pharmacists] would be aware of that they could then help me with*” [ID31]. Others saw the benefit in pharmacists’ involvement in PND screening, especially as it was “*an opportunity for someone to receive care… where you weren’t necessarily seeing the GP regularly but you’re seeing a pharmacist*” [ID17].

Most women felt that a proposed referral pathway from a pharmacist to a GP could be useful, explaining that “*I’d be more comfortable following up on the basis of someone else advising that… being able to walk into my GP saying, ‘Well, the pharmacist told me I have to come,’ can sometimes just make it a little bit more likely to happen*” [ID27]. However, this referral pathway may be only “*somewhat useful*”, as pharmacists “*can’t refer you to the psychologist*,” [ID38] and an additional referral to a GP is necessary to access subsidised specialist mental healthcare. Nonetheless, many still believed pharmacist-delivered PND screening and care to be an opportunity or “*a guide to go and speak to someone else about it, someone else who’s specialized in it*” [ID20]. This openness to pharmacist’s roles in perinatal mental health, provided referral pathways are established, can be considered a potential facilitator to pharmacist-delivered PND screening.

### 3.9. Theme 3: Pharmacist Visibility in PND Screening Care

Two subthemes: “pharmacist mental health training” and “service promotion”, were derived from the theme “pharmacist visibility in PND screening care.” The subtheme “pharmacist mental health training” was mapped to CFIR domain, “outer setting” and its associated constructs “patient needs and resources”. The subtheme “service promotion” was mapped to CFIR domain, “outer setting” and its associated construct “cosmopolitanism”. This “service promotion” subtheme was also mapped to CFIR domain, “process of implementation” and its associated construct “planning”.

### 3.10. Subtheme 3.1: Pharmacist Mental Health Training

Women felt that visibility and awareness of pharmacists’ mental health training in perinatal mental healthcare could facilitate patient comfort and confidence, explaining that “*If I knew at the big pharmacy that there was a specialist pregnancy or early childhood pharmacist… someone who specialises in that area, then I would definitely go and talk to that person*” [ID20]. Women also noted the need for pharmacies to actively increase the visibility of their work in PND to the public “*If there’s a pharmacy that has mental health accreditation, if they displayed [this accreditation] in their pharmacy… I’d trust them more. I’d be willing to come to them regarding my mental health issue… displaying those credentials are important*” [ID12].

### 3.11. Subtheme 3.2: Service Promotion

Women highlighted the importance of promotion to improve the visibility of pharmacist-delivered perinatal mental healthcare, in that pharmacies needed to provide “*more information, whether it be pamphlets or… advertising to say that they [pharmacists] do support… mental health and that they’re approachable, that you can discuss those sort of things with them*” [ID11]. For example, “*if you put a poster near the pharmacies… like ‘did you know that depression is common in pregnant women because of this… and you can ask your pharmacy about that?’ If I saw a sign like that, I would be more willing to ask them*” [ID12]. Furthermore, the need for promotion and support from other recognised perinatal care providers was highlighted as “*women trust their own GP or midwife much more in pregnant issues… so if they can refer the pharmacist or they can mention*, “*if you need help, you don’t always need to come to me*” *because sometimes it’s very difficult to book midwife… and sometimes GP as well*” [ID1].

Suggestions to improve pharmacist-delivered PND screening service acceptability and facilitate pharmacist-delivered PND screening included ensuring that the public was aware that such a service was a “*new protocol or new care initiative of the government*” [ID14]. Appropriately trained pharmacists and their services would subsequently be recognised as part of the perinatal mental health multidisciplinary system. The importance of support from the broader health system was crucial, as “*if I was given [the pharmacies’] details when I gave birth through the hospital, then I think I’d be more inclined to use that [pharmacy service]. I would feel like it’s supported*” [ID25].

### 3.12. Theme 4: Patient—Pharmacist Relationships

There were no subthemes derived from the theme of “patient–pharmacist relationships.” This theme was mapped to CFIR domain “process of implementation” and its associated construct “planning”. The theme was also mapped to the CFIR domain “characteristics of individuals” with associated constructs “individual identification with organisation”, and domain “intervention characteristics” with construct “relative advantage”.

Women’s perceptions of pharmacist-delivered PND screening were often informed by their personal experiences with pharmacists who were “*well qualified, approachable, easy to talk to and quite knowledgeable*” [ID11], as well as “*very supportive, quite friendly*” [ID13]. Women explained that pre-existing relationships with pharmacists were important in promoting comfortability and engagement with pharmacist-delivered mental healthcare “*I’d feel comfortable if it was a familiar pharmacist… uncomfortable, if it wasn’t a pharmacist that I was familiar with*” [ID 11]. Without existing rapport with their pharmacist, some women expressed they “*wouldn’t be willing to just have a sudden conversation with them [pharmacists]*” about PND [ID31]. Women also reported “*trust in their [pharmacists’] ability and trust that I won’t get turned away*” [ID15]. Overall, positive personal interactions with pharmacists may facilitate pharmacist-delivered PND screening.

Pharmacists were perceived as specialists in medication and “*in certain areas, more knowledgeable than GPs… so I’m very comfortable going to a pharmacist…I think if you’ve already got that trust for other issues, you would have trust in them for depression or any of those mental health needs*” [ID37]. Conversely, others felt their interactions with pharmacists were “*transactional*” [ID4] and reported that they “*don’t really see pharmacists as a place to go for mental health*” [ID12]. In relation to perinatal mental healthcare, some explained that they “*probably wouldn’t be willing to see a pharmacist… because I see them more as someone that can help me with medication… I wouldn’t necessarily see them as someone that would be knowledgeable in mental health…because… my relationship with my pharmacist is really transactional*” [ID31]. Furthermore, some felt that pharmacists’ passive role in the pharmacy contributed to this perception: “*a lot of pharmacists are… in the back. Not really seeing them doesn’t make it very accessible*” [ID15]. The lack of rapport between a pharmacist and their patient is a possible barrier to pharmacist-delivered PND screening delivery.

Conversely, others noted the potential benefit of anonymity with a pharmacist not known to the woman if “*they [women] actually have a good relationship with their GP, they might actually feel a bit embarrassed or not comfortable… going to see a pharmacist who you don’t know, you might be more willing to bring [up] anything*” [ID25]. Similarly, the preference for anonymity was explained by others, in that “*if there is no disclosure of name or personal details, I’ll be fine filling it [screening tool] out*” [ID7].

Participants provided various strategies to facilitate acceptability of pharmacist-delivered PND screening and care. For example, the importance of “*rapport*” [ID34] was highlighted, as was how pharmacists initially approach women so that “*it doesn’t feel … invasive and that it is relevant*” [ID34]. With appropriate training and increased visibility, pharmacists could build on their trust with women to become “*an effective point to screen people … if they notice that I’m struggling, they ask me some questions and then they could point me to the right direction. I would really appreciate that*” [ID12]. Furthermore, it was important to women that “*the right empathy is being shown, it shows that they are concerned for me and I’ll be acceptable and accepting [sic] to be able to speak more with the pharmacist*” [ID8].

### 3.13. Theme 5: Factors Influencing Service Accessibility

Three subthemes: “PND screening funding”, “appropriate approaches towards PND screening” and “accessibility of pharmacist-delivered PND screening”, were derived from the theme “factors influencing service accessibility.” Subtheme “PND screening funding” was mapped to CFIR domain “intervention characteristics” associated with constructs “cost”, “design quality and packaging”, as well as “relative advantage”. Subtheme “appropriate approaches towards PND screening” was mapped to domain “process of implementation” associated with construct “planning”. Subtheme “accessibility of pharmacist-delivered PND screening” was mapped to CFIR domain “intervention characteristics” associated with construct “relative advantage” and domain “characteristics of individuals” associated with construct “knowledge and beliefs about the intervention”.

### 3.14. Subtheme 5.1: PND Screening Funding

Although some women were willing to pay for a pharmacist-delivered PND screening service, most preferred the service to be bulk-billed, free or covered by Medicare. Those who were willing to pay for the service quoted prices ranging from AUD 10–150, costs that would typically reflect the cost of a GP co-payment in their area and the expectations of what a pharmacist-delivered PND screening service would encompass. Women who were willing to pay for the service explained “*I don’t think you can put a price on your mental health… I’d definitely be happy to pay a little bit out of pocket for it. I’d hope that service would be bulk billed so it’s family friendly and affordable so people can access that service*” [ID11].

Women that were unwilling to pay for the service explained that they “*don’t pay for it [screening] anywhere else*” [ID31]. Having costs incurred at every healthcare professional consultation, including the pharmacist-delivered PND screening service, could become “*very expensive*”, but women noted that “*if the pharmacist could prescribe medication and it was an alternative to seeing the GP, then yeah, I’d be happy to pay for it*” [ID30]. Women’s perceptions of pharmacists’ roles also impacted their perceived value of a pharmacist-delivered PND screening service “*I don’t think you should be paid because it’s not a private practice. It’s not necessarily psychology background or any kind of medical background per se to have that capacity to charge for the consult. So, it’ll probably have to be some community based initiative*” [ID6]. Perceived value of the PND screening service also depended on what would be provided by the pharmacist, in that “*if they’re a specialist pharmacist, or they’re providing extra information that I don’t know already, then that’s a value to me. I would pay for that*” [ID20]. However, participants also noted that out-of-pocket costs could compromise accessibility, thereby presenting a potential barrier to pharmacist-delivered PND screening implementation, as “*if you are just approaching pregnant women… to catch the gap of people who wouldn’t have gone the doctor… you want to tap into those who didn’t think there’s anything wrong with them… and you don’t really want to penalise that*” [ID15].

### 3.15. Subtheme 5.2: Appropriate Approaches towards PND Screening

Barriers including lack of privacy and time constraints were concerns raised regarding community pharmacist-delivered PND screening. Women suggested solutions including private, sit-down consultation rooms, explaining that “*if a community pharmacy perhaps had a service where they had a private consultation room, were able to sit down with you and then actually go through the screening questionnaire and what it means for you and the repercussions of the results… I think that would actually be quite helpful*” [ID13]. Without a private consultation room, women felt that perinatal mental health would be “*quite difficult for people to bring up*” [ID29]. Approaches to conducting PND screening included “*being able to make an appointment and book in advance*” [ID6], as well as suggestions to “*start off being a bit of an informal chat down an aisle*” [ID34] prior to the private consultation. Another consideration was to ensure that screened women are comfortable during the screening process, with thoughts as to “*how long it’s [PND screening] going to take, because they’re [PND women] not going to want to stand there for 10, 15 min talking… their feet might hurt… because you’re just carrying so much extra weight*” [ID20], with a seated area being preferred.

### 3.16. Subtheme 5.3: Accessibility of Pharmacist-Delivered PND Screening

Cost and convenience were barriers to visiting one pharmacy regularly, with women stating that “*I just go to the pharmacy wherever [sic] I’m near. So if I’m going to the shops somewhere and there’s a pharmacy near[by], that’s the one I would go to*” [ID10]. Despite this, pharmacist-delivered PND screening service was perceived by women to have advantages in terms of its accessibility. In comparison with other healthcare professionals, including GPs, women reported that pharmacists were “*available more than doctors… you can go into a pharmacy, wait 10–15 min and see someone pretty much any time…*, *whereas doctors, you might have to wait weeks to get in*” [ID37]. Similarly, women recognised that “*some people don’t see an obstetrician. They might not get these screening questions*” [ID9] and **“***where they can’t really get good access to the midwife, then maybe it [pharmacist-delivered screening] would be useful*” [ID12]. Women also acknowledged that they often visit their “*pharmacist a lot more than you’re seeing the doctors*” [ID30]. As a result, “*changes [would be] be picked up on far faster, which, of course, with depression and things is what you need*” [ID22].

The pharmacist-delivered PND screening service was also viewed as an “*extra layer of support*” [ID19], a “*catchment system, catching the people that might otherwise fly under the radar… it would be beneficial overall for both the mother, the wider support system and the baby*” and “*streamline the [healthcare] process*” [ID40]. However, it was noted that the screening should not feel too repetitive and that it would be acceptable “*[As long as I ] didn’t feel that I was getting screened every three minutes*” [ID30], since women should have already been screened through other pathways and may not feel that pharmacist-delivered PND screening “*was needed*” [ID31].

## 4. Discussion

This study is the first to use the CFIR framework to explore perinatal women’s views and attitudes towards and the acceptability of community pharmacist-delivered PND screening and care. Facilitators included improving the visibility of pharmacist expertise and training, developing strong patient–pharmacist relationships as well as improving comfortability and ensuring privacy during patient consultations. Barriers included time constraints and cost. Pharmacist-delivered PND screening could contribute to early detection and intervention, thereby supporting existing perinatal mental healthcare infrastructure.

By exploring CFIR domains, ‘intervention characteristics’, ‘characteristics of individuals’ and ‘process of implementation’, this study revealed that pharmacists’ visibility as part of the mental healthcare team, women’s perceptions of pharmacists’ training in mental health and the patient–pharmacist relationship influence pharmacist-delivered PND screening acceptability and patient comfort, as well as willingness to engage with a pharmacist-delivered PND screening service. This is consistent with previous studies demonstrating that patients’ perceptions of pharmacists’ expertise impact patient–pharmacist relationship quality, satisfaction and relationship commitment [47]. Despite the fact that an increasing number of pharmacy graduates are equipped with mental health training, whereby 90% of Australian pharmacy program providers are already providing or intending on providing MHFA training to students [42], many participants viewed pharmacists’ scope of practice as being exclusive to medications, medication safety and related advice. Studies show that factors which reduce patients’ trust in pharmacists include patients’ lack of awareness of a pharmacist’s scope of practice, professional qualification and regulation, variability in pharmacist competency, transparency in remuneration and community pharmacies’ business positioning [48]. Consideration of more tailored and effective marketing of existing and emerging pharmacist roles in public health [49], including PND screening, is required to increase public awareness of pharmacists’ roles in health promotion. In addition to improving the awareness of pharmacists’ roles in perinatal mental health, standardisation of care across pharmacies is also crucial in improving service acceptability and patient comfort. Gregory and Austin identified that inconsistencies in patients’ experiences across pharmacies was a trust-diminishing factor [48]. Therefore, ensuring that all pharmacists received standardised validated training and that the public is aware of this training consistency can improve trust.

Interestingly, some factors that influence acceptability, identified by women in the current study, have also been echoed by community pharmacists in previous studies [16], with stakeholders recognising that patient trust, privacy, time for screening and pharmacist training can facilitate service provision. Community pharmacists also recognised that support from other primary health providers and remuneration as integral to successful service delivery [16]. Additionally, obstetrics and gynaecology providers highlighted that improved collaboration and communication with, as well as training for community mental health providers and pharmacists, would facilitate timely perinatal pharmacotherapeutic treatment for patients [50]. Although pharmacists’ expanded scope of practice into areas such as prescribing have gained support by doctors and patients in countries such as Scotland [51], these attitudes may not be widely supported in Australia [52]. Subsequently, research into other primary care provider’s views towards and acceptability of pharmacist-delivered PND screening is also necessary. Given the ever-increasing demand for preventative care and treatment in primary care settings, pharmacists potentially have the capacity to take on extended roles in primary care [53], especially when supported by an interprofessional team [54].

There is emerging evidence around the feasibility and effectiveness of pharmacists’ roles in depression screening [55], with peak pharmacy bodies also supporting pharmacists’ roles in the screening and early detection of mental illness [56]. By exploring the CFIR domain of “intervention characteristics”, increased contact with healthcare professionals such as pharmacists through pharmacist-delivered PND screening services was found to assist in increasing PND care accessibility. In fact, on average, annually, Australians make 18 visits to pharmacies [57], where 40.7 million mental health prescriptions are dispensed [58]. Such frequent interactions with pharmacists provide opportunities for perinatal women to have conversations about their mental health, particularly in non-metropolitan areas where access to perinatal mental healthcare may be lacking. This may be, in part, due to specialist health workforce shortages in rural and remote areas, resulting in an increased reliance on primary care professionals to provide these health services [59]. Specifically, pharmacists can support existing perinatal mental health providers in effectively identifying, caring and referring at-risk women. Although some evidence suggests that there is a higher prevalence of antenatal depression in urban populations with no significant difference in postnatal depression prevalence between urban and rural populations in Australia [60], evidence from the United States and United Kingdom has demonstrated an increased risk of developing PND in rural populations [61,62]. With 1 in 5 women still not screened for PND [7], this study affirms that women see the value of pharmacists screening, especially when 20% of women do not receive help for PND [6]. Therefore, increased contact with healthcare professionals such as pharmacists through pharmacist-delivered PND screening services may assist in increasing PND care accessibility.

## 5. Strengths and Limitations

This study provides important insights into stakeholders’ views of pharmacist-delivered PND screening and includes participants from a broad geographical distribution across Australia. Nonetheless, the method of recruitment may have introduced bias, in that participants who were interested in mental health research may have been more likely to participate. Participants were aware that the interviewer was a pharmacist and may not have been comfortable sharing their true views on a pharmacist-delivered service. However, the varying attitudes resulting from this study have demonstrated that this factor would have been unlikely to influence the study’s validity. Furthermore, this study only explored attitudes among one stakeholder group, and studies including other stakeholders, namely pharmacists and other healthcare professionals involved in PND screening and care, will allow for triangulation and are needed to provide a more comprehensive understanding of prospective pharmacist-delivered PND screening service implementation. Existing studies have focused on pharmacists’ attitudes towards pharmacist-delivered PND screening services [16] and women’s attitudes towards other healthcare professionals’ roles in PND screening [19]. Hence, this study fills a gap in the literature pertaining to stakeholder views of PND screening in primary healthcare settings.

## 6. Conclusions

Exploring the attitudes of women towards pharmacist-delivered PND screening is crucial in developing PND screening services that are relevant, appropriate, acceptable and fulfil the needs of women. Most women felt that such services would be acceptable if trained pharmacists’ roles in mental health had improved visibility and support from other healthcare professionals. On the other hand, a lack of privacy, time constraints and potential out-of-pocket costs were identified as barriers to service acceptability and accessibility. By proactively engaging with women, pharmacist-delivered PND screening has the potential to increase screening rates in line with clinical practice guidelines, as well as facilitate early detection and intervention, thereby potentially contributing to improved maternal and infant health outcomes.

## Figures and Tables

**Table 1:** CFIR domains in relation to pharmacist-delivered PND screening [25,44,45].

Domain	Adapted Definition [25,44,45]
Outer setting	Economic, political, and social contexts of community pharmacy influencing implementation including women’s needs and resources, barriers and facilitators affecting meeting those needs, cosmopolitanism, external policy and incentives.
Characteristics of individuals	Women’s knowledge and beliefs as well as individual stage of change regarding the proposed pharmacist-delivered PND screening service. Individual identification with community pharmacy and perinatal care, self-efficacy and other personal attributes affecting implementation are considered.
Process of implementation	Women’s perceptions on how to plan, engage and execute effective pharmacist-delivered PND screening.
Inner setting	Women’s personal culture and readiness for PND screening implementation as well as the implementation climate, structural characteristics, networks and communications PND screening will be applied to.
Intervention characteristics	Aspects of pharmacist-delivered PND screening that influence its successful implementation including its relative advantage, complexity, cost, design quality and packaging.

**Table 2:** Characteristics of women interviewed (*n* = 41).

Characteristics	Frequency [n]	Percent [%]
Location (*n* = 41)		
New South Wales	20	48.8
Queensland	9	22
South Australia	5	12.2
Western Australia	3	7.3
Tasmania	3	7.3
Victoria	1	2.4
Age (*n* = 41)		
21–25	1	2.4
26–30	7	17.1
31–35	21	51.2
36–40	11	26.8
41–45	0	0
46–50	1	2.4
Multiparity (*n* = 41)		
First-time mothers	11	26.8
1 additional child	24	58.6
2 additional children	5	12.2
3 additional children	1	2.4

## Appendix A

**Table A1:** Standards for Reporting Qualitative Research (SRQR) * http://www.equator-network.org/reporting-guidelines/srqr/ (accessed on 16 October 2022).

Title and Abstract	Page/Line No(s).
**Title**—Concise description of the nature and topic of the study Identifying the study as qualitative or indicating the approach (e.g., ethnography, grounded theory) or data collection methods (e.g., interview, focus group) is recommended	Page 1
**Abstract**—Summary of key elements of the study using the abstract format of the intended publication; typically includes background, purpose, methods, results, and conclusions	Page 1
**Introduction**	
**Problem formulation**—Description and significance of the problem/phenomenon studied; review of relevant theory and empirical work; problem statement	Page 1–2
**Purpose or research question**—Purpose of the study and specific objectives or questions	Page 2
**Methods**	
**Qualitative approach and research paradigm**—Qualitative approach (e.g., ethnography, grounded theory, case study, phenomenology, narrative research) and guiding theory if appropriate; identifying the research paradigm (e.g., postpositivist, constructivist/interpretivist) is also recommended; rationale **	Page 2–4
**Researcher characteristics and reflexivity**—Researchers’ characteristics that may influence the research, including personal attributes, qualifications/experience, relationship with participants, assumptions, and/or presuppositions; potential or actual interaction between researchers’ characteristics and the research questions, approach, methods, results, and/or transferability	Page 2–4
**Context**—Setting/site and salient contextual factors; rationale **	Page 2–3
**Sampling strategy**—How and why research participants, documents, or events were selected; criteria for deciding when no further sampling was necessary (e.g., sampling saturation); rationale **	Page 3
**Ethical issues pertaining to human subjects**—Documentation of approval by an appropriate ethics review board and participant consent, or explanation for lack thereof; other confidentiality and data security issues	Page 3
**Data collection methods**—Types of data collected; details of data collection procedures including (as appropriate) start and stop dates of data collection and analysis, iterative process, triangulation of sources/methods, and modification of procedures in response to evolving study findings; rationale **	Page 3
**Data collection instruments and technologies**—Description of instruments (e.g., interview guides, questionnaires) and devices (e.g., audio recorders) used for data collection; if/how the instrument(s) changed over the course of the study	Page 3
**Units of study**—Number and relevant characteristics of participants, documents, or events included in the study; level of participation (could be reported in results)	Page 4–5 (in results)
**Data processing**—Methods for processing data prior to and during analysis, including transcription, data entry, data management and security, verification of data integrity, data coding, and anonymization/de-identification of excerpts	Page 3
**Data analysis**—Process by which inferences, themes, etc., were identified and developed, including the researchers involved in data analysis; usually references a specific paradigm or approach; rationale **	Page 3–4
**Techniques to enhance trustworthiness**—Techniques to enhance trustworthiness and credibility of data analysis (e.g., member checking, audit trail, triangulation); rationale **	Page 3–4
**Results/Findings**	
**Synthesis and interpretation**—Main findings (e.g., interpretations, inferences, and themes); might include development of a theory or model, or integration with prior research or theory	Page 4–10
**Links to empirical data**—Evidence (e.g., quotes, field notes, text excerpts, photographs) to substantiate analytic findings	Page 4–10, Appendix C
**Discussion**	
**Integration with prior work, implications, transferability, and contribution(s) to the field**—Short summary of main findings; explanation of how findings and conclusions connect to, support, elaborate on, or challenge conclusions of earlier scholarship; discussion of scope of application/generalizability; identification of unique contribution(s) to scholarship in a discipline or field	Page 10–11
**Limitations**—Trustworthiness and limitations of findings	Page 11–12
**Other**	
**Conflicts of interest**—Potential sources of influence or perceived influence on study conduct and conclusions; how these were managed	Page 12
**Funding**—Sources of funding and other support; role of funders in data collection, interpretation, and reporting	Page 12

* The authors created the SRQR by searching the literature to identify guidelines, reporting standards, and critical appraisal criteria for qualitative research; reviewing the reference lists of retrieved sources; and contacting experts to gain feedback. The SRQR aims to improve the transparency of all aspects of qualitative research by providing clear standards for reporting qualitative research. ** The rationale should briefly discuss the justification for choosing that theory, approach, method, or technique rather than other options available, the assumptions and limitations implicit in those choices, and how those choices influence study conclusions and transferability. As appropriate, the rationale for several items might be discussed together [63].

## Appendix B. Interview Guide

I would like to start by asking you a few questions about yourself.

1. What is your age?
2. Which Australian State or Territory do you currently reside in?
  Thank you. We are interested in the views of women who are currently pregnant or have recently had a baby. For the purpose of this interview, the perinatal period includes pregnancy and up to 12 months after giving birth.
3. Are you currently pregnant or have you given birth within the last 12 months?
  a. Is this your first experience of pregnancy/being a mother?
    - IF NOT: How many children do you have?
4. If you were to experience symptoms of depression during pregnancy or after giving birth, such as, low mood, loss of pleasure in activities that are usually enjoyable or irritability, would you feel comfortable to seek support?
  b. Where would you go to seek support?
    - Why would you go there/to this person?
5. Have you ever head of the term “perinatal depression”?
  *The next few questions relate to perinatal depression screening. Screening refers to the early detection of people who are at risk of an illness or health-related problem. For this study, we are enquiring about screening for perinatal depression. This may include the use of a questionnaire or other type of assessment regarding your mood. This may be completed with a healthcare professional, such as your GP, nurse or midwife during antenatal or postnatal care visits.*
  *Do you have any questions?*
  [If yes, respond to query; if not, then continue with questions]
  - Have you ever been screened for depression during your pregnancy and/or in the 12 months following delivery?
    **IF YES:**
    *Ask the person to elaborate if they remember*
    a. Did you complete a screening tool/test/questionnaire?
      **IF YES**
      - Did you complete this yourself or with your health care professional?
      **IF with HCP**
      ii. Which health care professional?
    b. About how long did it take to complete?
    c. How did you feel about being screened?
    d. How important was it for this person (e.g., healthcare professional) to screen you?
    e. Were there any outcomes (e.g., follow-up, referral, treatment initiation) as a result of screening?
    f. When was it conducted (i.e., at what stage during pregnancy/postpartum)?
      - Was it only performed once?
        1. IF more than once: How many times?
    **IF NO**: Move on to question 6
6. Do you have a regular pharmacy that you go to for your medication and health needs?
  **IF YES:**
  a. Why do you go to this pharmacy specifically?
  b. Do you usually interact with a specific pharmacist when you go there?
  c. How would you describe your relationship with this pharmacist?
  **IF NO:**
  a. Is there any reason why you don’t have a regular pharmacy?
  b. Have you ever had a regular pharmacist who you sought advice from?
7. Are you aware of any health services that are available for pregnant and postpartum women in community pharmacies?
  a. Have you ever used these services?
  b. What type of mental health care and services would you be willing to see a pharmacist about during pregnancy and/or in the postpartum period?
    - **IF NONE:** Why not?
8. Do you think mental health care is part of the pharmacist’s role for pregnant and postpartum women?
  d. Why or why not?
  e. What are some factors that might make it more acceptable for pharmacists to have a larger role in mental health care for pregnant and postpartum women?
  *The final few questions relate to the acceptability of perinatal depression screening in community pharmacy. Acceptability refers to how pleasing, agreeable or welcome an activity is to you, it can also refer to how willing or comfortable you might be to engage with a new service.*
  **Interviewer to state one of these statements based on the response to Question 5:**
  *You have stated that you have a regular pharmacist that you see when you need medication and health advice*
  **OR**
  *You have stated that you do not have a regular pharmacist that you see when you need medication and health advice and attend whichever pharmacy is convenient at the time…*
9. Imagine the next time you attend the pharmacy, the pharmacist noticed that you were pregnant/had recently given birth and asked you if they could have a conversation with you about how you are feeling or coping
  a. How would this make you feel?
10. Would it be acceptable for your pharmacist to approach you about your mental health and screen you for depression during pregnancy or in the postpartum period?
  i. Why or why not?
  ii. IF NOT ACCEPTABLE: What would make it more acceptable?
11. What if the pharmacist requested that you fill out a short screening questionnaire such as this one [show participant the Edinburgh Postnatal Depression Scale/ ask participant to refer to the scale that has been emailed to them in advance]
  b. How comfortable would you feel being screened for perinatal depression by your pharmacist in the pharmacy?
    - Why or why not?
  c. How useful would it be for your pharmacist to screen you for perinatal depression in the pharmacy?
    - Why or why not?
  d. How helpful would it be for your pharmacist to screen you for perinatal depression in the pharmacy?
    - Why or why not?
  e. Do you think pharmacist-led screening for depression using such a questionnaire is a good idea?
    - Why or why not?
  f. How willing would you be to be screened by the pharmacist and consider the pharmacist’s advice based on the results of this screening test (e.g., if the pharmacist recommended you saw the GP for further evaluation based on your score on the screening test)?
    - Why or why not?
12. If a perinatal depression screening and referral service were available in community pharmacy, would you be willing to pay for this service?
  g. Why or why not?
  h. IF WILLING: Is there a specific price that you would consider to be reasonable for such a service?
13. Is there anything else you would like to add?

## Appendix C

**Table A2:** Summary themes to pharmacist-delivered PND screening related to CFIR domains.

Themes	Sub-Themes	Related CFIR Domains	Related CFIR Constructs	Illustrative Quotes
Theme 1 Patient experience with existing PND support and screening services	Subtheme 1.1 Recognised sources of perinatal mental health support	Inner setting	Readiness for implementation Culture Networks and communications	“*I don’t see the point in struggling through something alone, and as much as you might have a wonderful supportive partner, parents, or friends, if they’re not professionals, they might not always give the best advice, or know the best thing to do so having the ability to get that support and advice from someone who is trained appropriately is really important*,” [ID5] “*I guess will be family and friends to begin with… they’re closest to me, they would understand my situation the best and wouldn’t really judge me*,” [ID9] “*Initially probably friends and family because they’ve had their own children and they could have all been going through the same things as myself, so they would probably have first-hand experience… with the GP, that’s… professional help, they could just give you some general advice or maybe direct you… to go talk to a counsellor… And then with the mothers group… I’m sure there’s other mums that are going through something either similar or something that they might have gone through in the past and they can share their experience or their advice*,” [ID18]
	Subtheme 1.2 Previous experience with PND screening	Outer Setting Inner Setting	Patient Needs and Resources Implementation climate	“*they [midwives] give very general information but there is a time limit* “*[…]*” *they told me to go to a private session if you need more information or you need more help*,” [ID2]. “*I remember my first pregnancy… at every visit, they had me fill out the mental health screening. This pregnancy, not once*,” [ID21] “*I didn’t have a problem with it at all… I think it’s good even if I don’t feel personally that I have anything wrong with me at the time. I appreciate that it’s a useful tool and it’s good to have a baseline anyway*,” [ID3] “*The first time I thought it was a bit odd. It was the first time that I’d ever seen something like that and I thought… some of these questions are really odd… After doing it a few times… I felt okay answering questions like that and see the real importance in it*…” [ID14] “*I thought it was good that I was doing it. I think it can be confronting, especially if you have been struggling a little bit, but I think shining a light on things when you’re not feeling right is a really positive thing to do*,” [ID5] “*Honestly, I think it’s just a box ticking exercise for the hospital… I’m not sure that anyone actually even reads it*,” [ID13] “*[I felt] indifferent… it made me realise that such problems could exist during pregnancy and sometimes you don’t realise if you have those kind of thoughts or symptoms*,” [ID9]
Theme 2 Familiarity with pharmacists’ roles	Subtheme 2.1 Awareness of pharmacist-delivered health services	Characteristics of individuals	Knowledge and beliefs about the intervention	“*I think, as you can weigh your baby… that’s about the only thing I really know*,” [ID27]
	Subtheme 2.2 Pharmacists’ roles in mental health		Individual identification with the organisation	“*For me, pharmacy here is a place where you got to go buy medicines… so I’m not sure how the pharmacy or the pharmacist plays a role in mental health*,” [ID7] “*I don’t really see pharmacists as a place to go for mental health, maybe more medication advice… but having said that… they meet a lot of… pregnant women… I think they can be quite an effective point to screen people*,” [ID12] “*I wouldn’t expect them to have that information [regarding PND]. If they did give it to me, I’d be interested in it. I just wouldn’t expect them to have it*,” [ID20] “*I’ve never thought of that as a role for a pharmacist but why not?*” [ID21] “*I’d like to think, as healthcare professionals, that they [pharmacists] play a role in identifying things* [PND],” [ID36]
Theme 3 Pharmacist visibility in PND screening care	Subtheme 3.1 Pharmacist mental health training	Outer Setting	Patient needs and resources	“*I think maybe having one person in the pharmacy that’s trained in mental health, just having somebody with a qualification makes it a lot stronger to have that around, or even just a community services certification so that they do know how to work with these mums*,” [ID32]
	Subtheme 3.2 Service promotion	Process of implementation	Cosmopolitanism Planning	“*I think maybe more awareness possibly. Even information packs that could be given to women in hospital when they leave*,” [ID22] “*Maybe if there was a section in the blue book where there was a role for the pharmacist to complete you would feel like it was a more… standard thing to do*,” [ID13] “*If there was some pamphlets… posters… just to say, ‘Hey, we’re here for you if you want to have a chat.’… it’s something that shows that you can speak to them [pharmacists] about anything.*” [ID18]
Theme 4 Patient—pharmacist relationships		Intervention characteristics Characteristics of individuals Process of Implementation	Relative advantage Individual identification with organisation Planning	“*I find them really helpful… Generally, they’ll either answer the questions that I have or… send me to somewhere that might be able to give me the answer… They’re all quite genuine and quite happy to stop whatever they’re doing and come and help you where you need it*,” [ID22] “*He’s [the local pharmacist] really… well qualified… approachable, easy to talk to and quite knowledgeable*.” [ID11] “*I think they’re fairly approachable, and I certainly would ask questions if I needed advice regarding medications or health concerns*,” [ID27] “*It’s [my relationship with the pharmacist] a non-relationship. It’s just, good morning, hello, how are you? Here’s a script. Thank you, bye*,” [ID23] “*[you interact with] store people more than you would speak to a pharmacist*,” [ID26] “*I would describe it [relationship with pharmacist] as… a transactional relationship*,” [ID30] “*I don’t have an issue but I know maybe some people will be more sensitive. It just depends how close you are with that pharmacist… But if you have that relationship with them where you have been getting medication from their pharmacy quite regularly, that same person, then I think it will be okay. I’d be quite comfortable with that*,” [ID9] “*If somebody had a relationship with their pharmacist where they speak to them regularly about their health or whatever, then I guess it could be some part of a conversation*,” [ID24]
Theme 5 Factors influencing service accessibility	Subtheme 5.1 PND screening funding	Intervention characteristics	Cost Design quality and packaging Relative advantage	“*I’m not sure that I’d be willing to pay for it because the GP is right there, and that’s free*,” [ID13] “*[I would] probably not [pay for PND screening]… then going to the GP and paying for the GP and then paying for… counselling suddenly becomes a very expensive game. If the pharmacist could prescribe medication and it was an alternative to seeing the GP… I’d be happy to pay for it*,” [ID30] “*I don’t pay for it anywhere else anyway*,” [ID31] “*[I am willing to pay] because I can afford to, but I would hate to think that people who needed the service missed out, or didn’t get the support they needed because they couldn’t afford it” [ID5]* “*Yes [I would pay]* “*[…]*” *if I felt like I needed it* “*[…]*” *and felt like it was a well thought out service*,” [ID25] “*Yeah [I would pay] if they [pharmacists] could do it* “*[…]*” *it’d be easier than a GP, more accessible and there wouldn’t be sick people around*,” [ID33]
	Subtheme 5.2 Appropriate approaches towards PND screening	Process of implementation	Planning	“*[I would be comfortable] as long as it’s [screening] delivered in a timely manner*,” [Participant 3] “*I think if it’s done in a quiet room somewhere, you’d still feel comfortable. It may start off being a bit of an informal chat down an aisle*,” [ID34] “*Maybe if they have… a dedicated area but somewhere where you can just like the pharmacist maybe can pull a customer to the side and just say, talk to them or* vice versa *if they want to have a chat with the pharmacist just quickly*,” [ID18] “*It’s harder for women to actually have that conversation is when you’re at a pharmacy, it’s quite public. It’s very open. You’re not in a small room where you can talk to your GP in private. So I can imagine it’s quite difficult for people to bring that [PND] up*,” [ID29]
	Subtheme 5.3 Accessibility of pharmacist-delivered PND screening	Intervention characteristics Characteristics of individuals	Relative Advantage Knowledge and beliefs about the intervention	“*[I go to this pharmacy for] Mainly convenience, although… I really like the pharmacist… but I feel like I’ve spoken to her on a number of occasions and built up that kind of relationship… she is someone that I’ve gone to in the past because I want some specific advice*,” [ID4] “*We have a community pharmacy which is probably 200 metres from my house… I think the couple that are in the pharmacy… they’ve got a baby. So I think all the mums particularly love going there because of that. Both the husband and wife are both pharmacists and they’re both just so lovely, very supportive*,” [ID13] “*It would be good because… you’re there [in the pharmacy] probably more often than… a lot of other places and being a local service, it’s quick and easy, accessible… and not as daunting as walking into a doctor’s office, or a clinical setting*,” [ID27] “*It’s one [pharmacy] that kind of has a membership that makes it slightly cheaper so we have a family membership that we can use at that particular one that’s local as well*,” [ID19] “*As a new mum, it would be good because you might not make the time to go and see anybody else, but you might be there [in the pharmacy] for other reasons*,” [ID26] “*It is a good idea because it is just another person who’s checking in on you… to have somebody checking in on you without you having to actively seek them out is helpful*,” [ID4] “*I think that it is really useful, and I think it’s just because it’s like one extra tool to sort of help*,” [ID32]
